# Applying Nonparametric Methods to Analyses of Short-Term Fine Particulate Matter Exposure and Hospital Admissions for Cardiovascular Diseases among Older Adults

**DOI:** 10.3390/ijerph14091051

**Published:** 2017-09-12

**Authors:** Louis Anthony (Tony) Cox, Xiaobin Liu, Liuhua Shi, Ke Zu, Julie Goodman

**Affiliations:** 1 Cox Associates Consulting, Denver, CO 80218, USA; tcoxdenver@aol.com; 2 Gradient, Cambridge, MA 02138, USA; xliu@gradientcorp.com (X.L.); LShi@gradientcorp.com (L.S.); kzu@gradientcorp.com (K.Z.)

**Keywords:** air pollution, fine particulate matter, epidemiology, causal analysis, nonparametric, cardiovascular disease

## Abstract

Short-term exposure to fine particulate matter (PM_2.5_) has been associated with increased risks of cardiovascular diseases (CVDs), but whether such associations are supportive of a causal relationship is unclear, and few studies have employed formal causal analysis methods to address this. We employed nonparametric methods to examine the associations between daily concentrations of PM_2.5_ and hospital admissions (HAs) for CVD among adults aged 75 years and older in Texas, USA. We first quantified the associations in partial dependence plots generated using the random forest approach. We next used a Bayesian network learning algorithm to identify conditional dependencies between CVD HAs of older men and women and several predictor variables. We found that geographic location (county), time (e.g., month and year), and temperature satisfied necessary information conditions for being causes of CVD HAs among older men and women, but daily PM_2.5_ concentrations did not. We also found that CVD HAs of disjoint subpopulations were strongly predictive of CVD HAs among older men and women, indicating the presence of unmeasured confounders. Our findings from nonparametric analyses do not support PM_2.5_ as a direct cause of CVD HAs among older adults.

## 1. Introduction

Over the past 50 years, considerable air pollution epidemiology research has focused on quantifying statistical associations between ambient concentrations of air pollutants and adverse health outcomes using concentration-response (C-R) regression models. For instance, substantial evidence has linked fine particulate matter (PM_2.5_) concentrations to cardiovascular disease (CVD) mortality based on these models [1,2]. However, whether such associations are supportive of a causal relationship has often been unclear, and few studies have employed formal methods of causal analysis [3,4].

Some investigators have causally interpreted the statistical associations between PM_2.5_ levels and mortality rates by assuming that reductions in ambient concentrations of air pollutants cause approximately proportional reductions in adverse public health consequences, and then estimating the consequent reductions in health risks from hypothetical reductions in air pollution levels (e.g., [5]). Other researchers also used this data-driven approach to draw causal conclusions from observational data by treating measures of association, such as relative risks and quantities derived from them (e.g., attributable risks, etiologic fractions, burden of disease estimates), as causal measures (e.g., [6]). One caveat with this concept is that a statistical association is not necessarily causal. If the association is at least partly attributable to modeling biases or to confounding factors (for example, poverty is associated with both living in more polluted areas, and mortality and morbidity; [7]), and if the combinations of factors (e.g., income, education, smoking) that actually cause increased mortality and morbidity are uncertain, then reducing pollution might not reduce mortality and morbidity rates as much as predicted by these models [7]. Another uncertainty is that the direction and magnitude of associations often depend on modeling choices, making conclusions based on them unreliable in general [3].

A more sophisticated approach to the causal interpretation of observational data makes assumptions about what would have happened if exposures had been different from their true values, and it attributes differences between what did happen and predictions about what would have happened to differences between the real and the hypothetical (“counterfactual”) exposures. Potential outcome models are typically used to carry out the needed calculations. These models usually depend on untested, and perhaps untestable, statistical modeling assumptions [8]: e.g., a proposed instrumental variable regression model is valid (i.e., one or more variables can be validly used as “instruments” to isolate the variations in exposure that are uncorrelated with the error) [8], seasonal confounders other than temperature (e.g., humidity, length of daylight) can be ignored [9], there are no unobserved confounders, or regression models accurately predict what would have been seen had exposure concentrations been different than they actually were. Although experts in causal analysis have warned that such assumption-based causal interpretations of observational data are not well justified (e.g., [10]), clearly better alternatives have been in short supply. 

Vibrant research communities in physics, economics, neuroscience, machine learning, and artificial intelligence have contributed important new ideas and algorithms for drawing causal inferences from non-experimental data and have compared the performance of different methods (e.g., [11]). Most of the top-performing methods use these alternative approaches, described in more detail in Section 2.4, of conditional independence and information principles, nonparametric analyses, and model ensembles. These approaches combine the basic idea of counterfactual models—that differences in causes make effects differ, with the related idea that differences in causes help to predict differences in effects—providing the advantage that observational data can be tested without assumptions about what would have been seen had exposures or other conditions been different from those observed. Empirically, results from a recent competitive evaluation of the performance of different causal inference algorithms [11] found that nonparametric regression tree methods led to a bias of −0.007 in estimating the causal effects from data, compared to a bias of −0.15 for a counterfactual algorithm, a root mean-squared error for predictions of 0.02 compared to 0.41 for the counterfactual algorithm, and much smaller uncertainty intervals and higher coverage probabilities. Thus, it appears that the nonparametric tree-based approach can complement counterfactual approaches in at least some cases by requiring fewer assumptions and providing better performance. 

By employing nonparametric approaches (e.g., Bayesian network and regression trees), the present study aims to examine associations between short-term exposure to PM_2.5_ and hospital admissions (HAs) for CVD among older adults (aged 75 years and older) in Texas, USA, along with possible causal interpretations. We focused on this subpopulation because they may be more susceptible to heart failure after short-term ambient PM_2.5_ exposure than younger adults [12,13]. Focusing on older adults also allowed us to compare the results with previous analyses that have used similar methodology for the Los Angeles, CA, USA air basin [14]. The goal of this work is to provide an alternative perspective, which can complement traditional epidemiological methods for addressing causality.

## 2. Materials and Methods 

### 2.1. Hospital Admission Data

Daily hospital discharge data were obtained from the Texas Department of State Health Services (TDSHS) for the years 2001–2013 for patients who were hospitalized in emergency or urgent care departments in Dallas and Harris Counties, Texas, with a primary diagnosis of all-type CVD (International Classification of Disease, 9th Revision [ICD-9] codes 401–405, 401–417, 420–438, 440–445, 447–449). We aggregated individual-level data to county-level daily counts of total CVD HAs, as well as CVD HAs for specific sex (male and female) and age groups (18–75 years, 75 years and older). The study was approved by the TDSHS Institutional Review Board (IRB) #1 in June 2016 (IRB# 16-011).

### 2.2. PM_2.5_ Data

Nationwide daily Federal Reference Method/Federal Equivalence Method (FRM/FEM) PM_2.5_ data (parameter code: 88101) for 2001–2013 were downloaded from the United States Environmental Protection Agency (US EPA) Technology Transfer Network (TTN) Air Quality System (AQS). County-level daily average PM_2.5_ concentrations were calculated from daily measurements at individual monitors.

### 2.3. Meteorological Data

Daily Quality Controlled Local Climatological Data (QCLCD) were downloaded from the National Oceanic and Atmospheric Administration (NOAA) for the years 2001–2013. We generated county-level daily average meteorological data, including daily minimum, maximum, and average temperature, and dew point temperature, based on FIPS county code (a five-digit Federal Information Processing Standard code that uniquely identifies counties and county equivalents in the US) in the QCLCD.

### 2.4. Statistical Analysis

We first examined C-R relationships between ambient PM_2.5_ concentrations and CVD HAs among older men and women (75 years and older) using parametric regression models (including multivariate linear regression, Poisson regression, and quasi-Poisson regression models), with adjustment for county; year; month; daily average, minimum, maximum and dew point temperature; and CVD HAs among older people of the opposite sex. We next employed various nonparametric methods to examine potential causality of the C-R relationships between PM_2.5_ and CVD HAs, using the following three key principles:

1. *Information principle*: Causes provide unique information that helps predict their effects and that cannot be obtained from other variables. (Conversely, if in some dataset the response R is conditionally independent of exposure concentration C, given the values of a covariate vector Z, then there is no evidence that C is a direct cause of R in that dataset.) This principle creates a bridge between well-developed statistical and machine learning methods for identifying informative variables that improve the prediction of dependent variables such as health effects, on the one hand, and the needs of causal inference, on the other. Only variables that help predict an effect by providing information that is not redundant with that from other variables (e.g., measured confounders) are candidates to be its causes. This constraint allows techniques of predictive analytics to be applied as a necessary condition for potential causation.
2. *Nonparametric analyses*: Most of the top-performing causal inference algorithms use multivariate nonparametric methods (most commonly, classification and regression trees) to identify information dependency relations among variables and to help avoid biases due to model specification errors. If no significant change occurs in the conditional empirical cumulative distribution function of a dependent variable as the value of an explanatory variable varies for any combination of values of the remaining variables (i.e., the dependent variable and the explanatory variable are conditionally independent), then the dependent variable is conditionally independent of the explanatory variable. This lack of dependence does not provide evidence that the explanatory variable is a cause of the dependent variable, because effects are not conditionally independent of their direct causes.
3. *Model ensembles*: Rather than relying on any single statistical model or nonparametric analysis, the top-performing causal analytics algorithms typically fit hundreds of nonparametric models (e.g., regression trees) to randomly generated bootstrap samples of the data and average the resulting predictions of how the dependent variable (e.g., income, education, smoking) depends on other variables. Such averaging over an ensemble of model results usually yields better estimates of how the dependent variable depends on other variables (e.g., pollutant concentrations) with lower bias and error variance than the estimates from any single predictive model.

Following these principles, we first quantified the associations between PM_2.5_ and CVD HAs among older adults in a partial dependence plot generated using the random forest approach. A random forest is a nonparametric model ensemble of hundreds of regression trees. Each regression tree segments the predictor space (the set of possible values of all the predictors) into a number of simple distinct and non-overlapping regions and uses the mean response of the training observations in a region to make a prediction for each observation in that region. By taking repeated random bootstrap samples from the training dataset, our Random Forest algorithm constructed 500 regression trees on each sample and averaged the resulting predictions. The partial dependence plot obtained a prediction of daily CVD hospitalizations from the random forest for each unique value of PM_2.5_ across its full range, accounting for the effects of the other variables. Plotting predicted CVD hospitalizations against PM_2.5_ yields a visualization of the partial effect of PM_2.5_ on CVD HAs in older adults. For the partial dependence plots of PM_2.5_ and CVD HAs in older adults, we conditioned on various covariates such as county, year, month, day, temperature, and dew point. In addition, we also conditioned on observed CVD admissions for control populations disjoint from those of interest (i.e., admissions among older people of the opposite sex and HAs among younger adults) in an attempt to control for unobserved confounders that affect all members of these populations. 

We also used a Bayesian network learning algorithm to identify information relations (conditional dependencies) among the response variable, CVD HAs of older men or women, and predictor variables including county, year, month, daily PM_2.5_ concentration, daily average temperature, maximum temperature, minimum temperature, dew point temperature, and all-aged CVD HAs for men and women. These variables were represented by nodes in a directed acyclic graph (DAG), and conditional dependencies were represented by arrows. Nodes that were directly connected by arrows (regardless of direction) represented variables that were not conditionally independent of each other, even after conditioning on all other variables [15]. The DAG structure of variables provides an important guide to potential causation [16], since only the nodes adjacent to a given node are identified as potential direct causes of that node (based on the principle that causes are informative about their effects), and it is well worth applying different algorithms to confirm or correct its main conclusions. 

All statistical analyses were conducted using the Causal Analysis Toolkit (CAT), a free add-in for Microsoft Excel developed by the George Washington University Regulatory Studies Center using R 3.2.5 [17].

## 3. Results

CVD HAs, ambient PM_2.5_ concentrations, and meteorological factors in Dallas and Harris counties, TX, USA, from 2003 to 2011 are presented in Table 1 and Table S1. There were a total of 775,576 adult CVD HAs (mean ± standard deviation [SD]: 84.4 ± 28.3 daily visits) in Dallas and Harris counties from 2003 to 2011, which included 389,072 (50.2%) females (mean ± SD: 42.3 ± 14.7 daily visits) and 386,415 (49.8%) males (mean ± SD: 42.1 ± 15.2 daily visits) (Table 1). A total of 241,567 (31.1%) were 75 years of age or older (mean ± SD: 26.3 ± 9.4 daily visits), among whom 149,557 (61.9%) were female (mean ± SD: 16.3 ± 6.3 daily visits) and 91,987 (38.1%) were male (mean ± SD: 10.0 ± 4.5 daily visits). The mean PM_2.5_ concentration for the two counties was 12.2 μg/m^3^ (range: 0.6–57.5 μg/m^3^). The average temperature was 69.1 °F (range: 18.0–100.0 °F), and the average dew point temperature was 55.6 °F (range: 5.5–77.3 °F). Correlations between environmental factors were generally weak but highly statistically significant (Table 2). PM_2.5_ is positively correlated with temperature and CVD HAs, except for in older males (r = −0.01, *p*-value = 0.49). 

**Figure 1:**
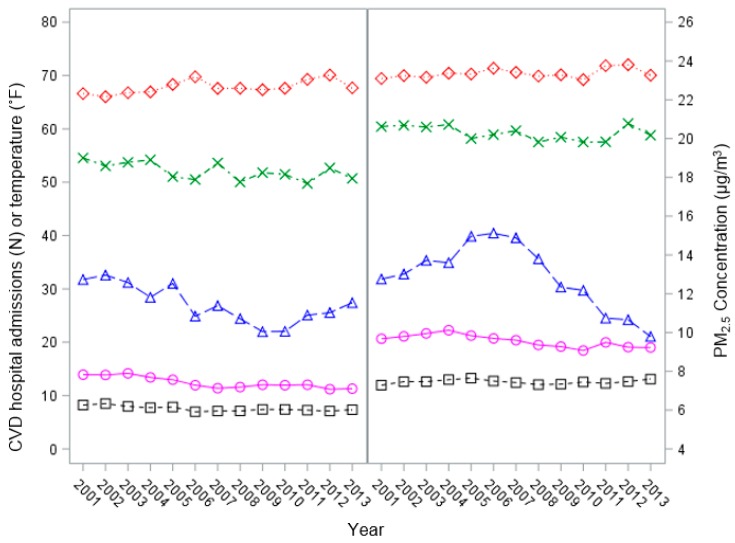
Annual average of daily PM_2.5_ concentrations, mean temperature, dew point, and daily CVD HAs for men and women, aged 75 years and older, in Dallas County (**left**) and Harris County (**right**), Texas, 2001–2013. Circles represent CVD HAs for women; squares represent CVD HAs for men; triangles represent daily PM_2.5_ concentrations; diamonds represent daily average temperature; crosses represent dew point.

The temporal trends for annual average daily CVD HAs among older adults, ambient PM_2.5_ concentrations, and meteorological factors in Dallas and Harris counties, Texas, between 2001 and 2013 are shown in Figure 1. Harris County was slightly warmer than Dallas County, and annual average daily temperatures increased in both counties from 2001 to 2013. In both counties in each year analyzed, older women (aged 75 years and older) had much higher daily HAs than older men. CVD HA risk also declined far more quickly among older women than among older men between 2001 and 2013 (with no decline among older men in Harris County). PM_2.5_ concentrations were in the range of 12 ± 2 μg/m^3^ in Dallas County for most years and were more variable in Harris County. In Dallas County, an overall decreasing trend was observed. In Harris County, however, PM_2.5_ concentrations fluctuated, rising from 12 μg/m^3^ in 2001 to 15 μg/m^3^ in 2006, before falling back to 10 μg/m^3^ in 2013.

Annual average daily CVD HAs for all adult men and women (aged 18 years and older), ambient PM_2.5_ concentrations, and meteorological factors are shown in Figure S1. CVD hospitalization rates declined for women and increased for men between the first few years and the last few years of this interval. Peaks and troughs in annual average PM_2.5_ do not correspond to peaks and troughs in annual average CVD HA rates in all women or older women (Figure 1 and Figure S1), and CVD HAs increased for men while decreasing for women (Figure S1). These observations suggest that factors other than PM_2.5_ might dominate causation of sex-specific CVD morbidity; otherwise, changes in PM_2.5_ would be correlated with corresponding changes in CVD HA rates. 

Monthly averages of environmental factors and CVD HAs are presented in Figure S2 (older men and women, aged 75 years and older) and Figure S3 (all adult men and women, aged 18 years and older). Finer temporal resolutions at months, instead of years, revealed that daily average temperature and daily dew point follow strong annual cycles in both counties. PM_2.5_ levels are more irregular, raising the possibility that effects of exceptionally high or low monthly average PM_2.5_ concentrations on CVD HAs should be detectable as deviations from their usual cyclical pattern throughout the year, if they exist. 

The associations between PM_2.5_ and CVD HAs in older adults estimated from three parametric models are presented in Table 3. When adjusted for CVD HAs for older adults of the opposite sex and all of the other meteorological covariates, a statistically significant positive association with PM_2.5_ is observed for CVD HAs in older women, but not in older men, from all three models (Table 3). 

**Figure 2:**
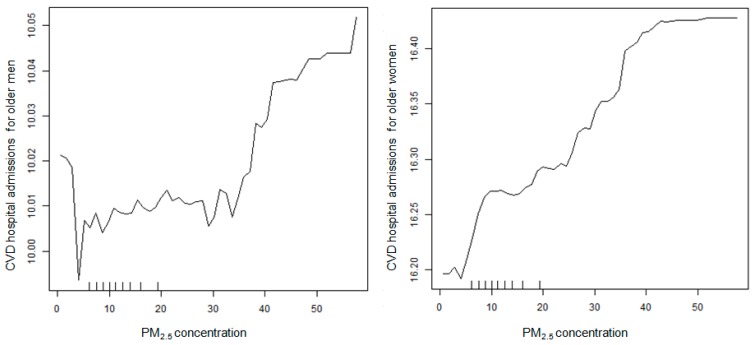
The partial effect of same-day PM_2.5_ on daily CVD HAs for men (**left**) and women (**right**), aged 75 and older. The partial dependence plots are based on random forests of 500 trees after conditioning on county, year, month, daily average temperature, minimum temperature, maximum temperature, dew point, and CVD HAs for disjoint control populations, including older people of the opposite sex and all people aged 18–75 years.

Using the nonparametric random forest approach, we assembled 500 regression trees based on 500 bootstrap samples and generated partial dependence plots to quantify the associations between daily CVD HAs among older adults and PM_2.5_ concentrations (Figure 2). For older men, there are about 10.01 ± 0.02 admissions per day, for days with PM_2.5_ concentrations ranging from less than 5 μg/m^3^ to over 50 μg/m^3^, after conditioning on other variables. Daily CVD HAs are higher for older men on most days with PM_2.5_ concentrations over 35 μg/m^3^ than on most days with lower PM_2.5_ concentrations, indicating a positive association between PM_2.5_ and CVD HAs. Supposing for purposes of a conservative quantitative risk assessment that this plot describes a genuine causal relation (rather than effects of unobserved, uncontrolled, or residual confounding), then the size of the effect would be less than a 0.1% increase in CVD HAs per day for older men per 10 μg/m^3^ increase in daily PM_2.5_ concentration. The analogous plot for women (right panel of Figure 2) shows daily CVD HAs of about 16.35 ± 0.10 and a less than 0.25% increase in CVD HAs per day per 10 μg/m^3^ increase in daily PM_2.5_ concentration. Therefore, compared to the results of parametric models, a positive but much weaker PM_2.5_-CVD HA association was observed from the partial dependence plots. However, daily CVD HAs in disjoint control populations (e.g., for older men, the control populations were older women and all people aged 18–75 years) may only be a crude approximation for the effects of unobserved confounders. Therefore, the weak positive association suggested by the plots does not fully support a causal interpretation. 

**Figure 3:**
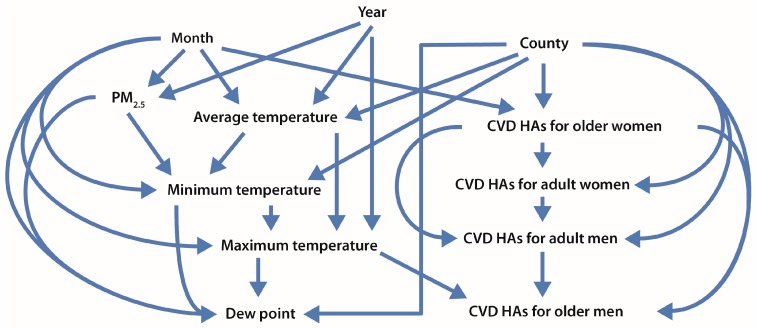
Conditional dependence relations among CVD HAs for older men and women (aged 75 years and older), adult men and women (aged 18 years and older), PM_2.5_, and other variables. The Bayesian network structure is generated from the primary dataset, including daily values of counts of CVD HAs for older men and women; counts of CVD HAs for adult men and women; daily PM_2.5_ concentrations, average temperature, minimum temperature, maximum temperature, and dew point as continuous variables; and county, year and month as categorical variables. An arrow between two variables, regardless of direction, indicates that they are conditionally dependent on each other.

We further examined the potential causality of the observed PM_2.5_–CVD HA associations using the nonparametric Bayesian network approach. Figure 3 shows the DAG structure of the variables discovered by a Bayesian network learning algorithm. Daily CVD HAs for older men depended directly on the county, daily maximum temperature, and daily CVD HAs for older women, as indicated by arrows between these three variables and daily CVD HAs for older men, respectively. Because CVD is not a contagious disease, we assume that CVD HAs for older men and older women do not directly affect one another (i.e., one should be conditionally independent of the other). Thus, the arrows between CVD HAs for older men and CVD HAs for older women, as discovered by the Bayesian network learning algorithm, are a result of unknown factors that can affect both of them, and CVD HAs for older women can be considered as a surrogate for these unknown factors. Similarly, daily CVD HAs for older women depend directly on county, the month of the year, and on unknown factors that also affect Daily CVD HAs for older men. From the figure, PM_2.5_ concentrations, temperature variables, and dew point depend on the month and year and also on each other. However, CVD HAs do not depend directly on PM_2.5_, since no arrow goes directly from PM_2.5_ to CVD HAs. Hence, PM_2.5_ is not identified as a direct cause of CVD Has; instead, CVD HAs appear to be conditionally independent of PM_2.5_, after conditioning on the other variables. 

Also shown in Figure 3, the month of the year is informative about PM_2.5_, on the one hand, and about CVD HAs for older women (directly) and CVD HAs for older men (indirectly, e.g., via paths that involve temperature variables), on the other. Hence, the month is a potential confounder of PM_2.5_-CVD HA associations for older men and women. Year, month, and PM_2.5_ are all informative about CVD HAs for older men (and hence CVD HAs for older women) through paths that involve temperature variables. Thus, PM_2.5_ can be significantly associated with CVD HAs for older adults, as seen in Table 3, even though it is not identified as a potential direct cause of them, since PM_2.5_ is not adjacent to any of the CVD variables in the DAG model.

**Figure 4:**
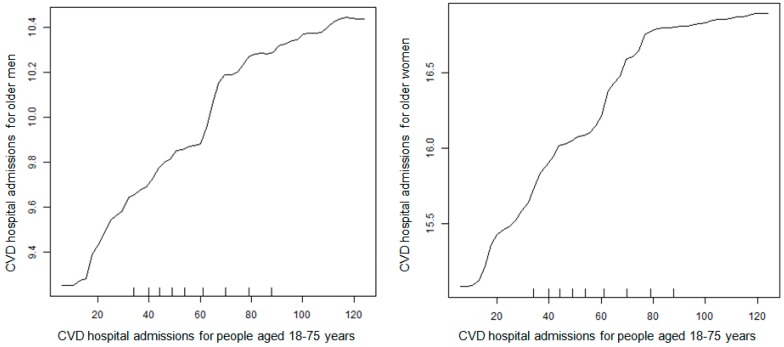
The partial effect of CVD HAs for people aged 18–75 years on CVD HAs for older men (aged 75 years and older, left) and older women (aged 75 years and older, right). The partial dependence plots are based on random forests of 500 trees after conditioning on county, year, month, daily average temperature, minimum temperature, maximum temperature, dew point, and CVD HAs for disjoint control populations, including older people of the opposite sex and all people aged 18–75 years.

**Figure 5:**
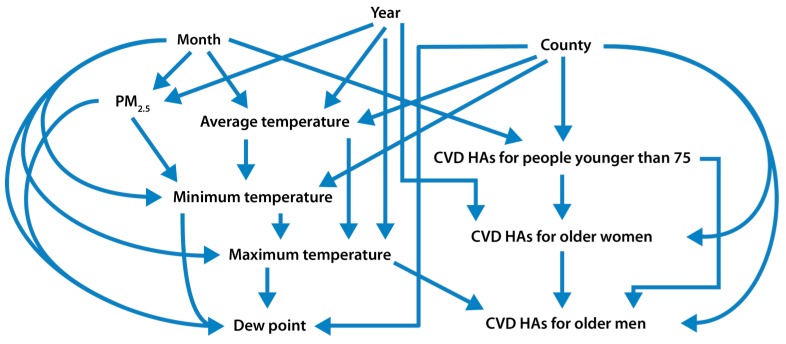
Conditional dependence relations among CVD HAs for older men and women (aged 75 years and older), people aged 18–75 years, PM_2.5_, and other variables. The Bayesian network structure is generated from the primary dataset, including daily values of counts of CVD HAs for older men and women; counts of CVD HAs for adult men and women; daily PM_2.5_ concentrations, average temperature, minimum temperature, maximum temperature, and dew point as continuous variables; and county, year and month as categorical variables. An arrow between two variables, regardless of direction, indicates that they are conditionally dependent on each other.

The partial dependencies for CVD HAs for older men (left) and older women (right) vs. CVD HAs among adults younger than 75 years are plotted in Figure 4. As CVD HAs among people younger than 75 years increased by about six-fold, CVD HAs for older men and women increased by about 12%, suggesting that common factors not included in the dataset affected both the younger and older adult population. Figure 5 shows the Bayesian network structure when CVD HAs among people younger than 75 years are included as a control. There are arrows between CVD HAs for people younger than 75 years and CVD HAs for older men (and women) in the DAG structure, again suggesting the existence of influential common factors. 

A single regression tree is also useful to identify variables that are informative for predicting CVD HAs among older men and women. In the regression trees fitted for CVD HAs among older men and women (Figures S4 and S5), county, month, and temperature variables were predictive of CVD HAs among older adults, but PM_2.5_ was not identified as a potential direct cause of CVD hospitalizations. These findings are consistent with the results from the Bayesian network in Figure 3 and Figure 5. 

The observation that CVD HAs for older men and women were not statistically independent of the CVD HAs in their respective disjoint control populations (or of each other) after conditioning on other covariates, as indicated by the arrows between them in Figure 3 and Figure 5, suggests that the month, year, county, and temperature are not the only important potential confounders of the association between PM_2.5_ and CVD HAs for older adults, i.e., unmeasured factors play a significant role in explaining observed dependencies. Thus, the positive associations in Figure 2 may be due to unobserved or incompletely controlled confounding by unknown factors that make CVD HA rates in disjoint subpopulations appear to be informative about each other in Figure 3 and Figure 5. CVD HAs among younger adults were linked to both PM_2.5_ (via multiple undirected paths) and CVD HAs among older men and women (directly), so unmeasured factors serve as a surrogate that could confound the observed partial dependence relation between them in Figure 2.

## 4. Discussion

We evaluated statistical associations and conditional dependencies between PM_2.5_ and CVD HAs in older adults, using both parametric and nonparametric methods. We found that PM_2.5_ is not well supported as a direct cause of CVD HAs among older men and women, although strong statistical associations between them were observed. This indicates that single C-R coefficients or other measures of association between air pollution exposure levels and health outcomes are not sufficient to draw conclusions about a causal relationship. The present study provides an example of how nonparametric analyses can complement traditional epidemiological methods and be used to identify potential causal relations among variables. 

Many time series and case-crossover studies have reported associations between short-term exposure to PM_2.5_ and increased CVD HAs. For instance, a US national study by Bell et al. [18] reported a 0.80% increase in CVD HAs (95% confidence interval [CI]: 0.59, 1.01%) among the Medicare population (over 65 years of age) per 10 μg/m^3^ increase in same-day PM_2.5_. Similar results were also derived in a meta-analysis by World Health Organization (WHO) region, with an overall summary estimate of 0.90% increase in all-age CVD HAs (95% CI: 0.26, 1.53%) per 10 μg/m^3^ increase in PM_2.5_ [19]. A recent study comprising 0.33 million Medicare beneficiaries found a larger increase of 1.88% (95% CI: 0.47, 3.31%) in CVD HAs for an interquartile range (IQR, 10.7 μg/m^3^) increase of PM_2.5_ [20]. Our quasi-Poisson regression models also found positive associations between PM_2.5_ and CVD HAs for both older men and women. The effect size for older women is similar to that reported by Bell et al. [20], with a 2.16% (95% CI: 1.05, 3.29%) increase per 10 μg/m^3^, while the result for older men is lower than previous studies, with a non-significant 0.15% (95% CI: −1.24, 1.55%) increase per 10 μg/m^3^.

The results of nonparametric (Bayesian network and regression tree) analyses, however, suggest that these increased risks may not indicate causation because other risk factors likely confound the observed associations. For example, although partial dependence plots indicate a non-linear positive association between PM_2.5_ concentrations and same-day CVD HAs after partially controlling for unobserved confounders, the statistical effect sizes are much smaller compared to the results of parametric models. For days with PM_2.5_ concentrations ranging from less than 5 μg/m^3^ to over 50 μg/m^3^, a less than 0.1% increase in CVD HAs for older men and a less than 0.3% increase in CVD HAs for older women per 10 μg/m^3^ increase in daily PM_2.5_ concentration were observed. Such small effect sizes are not sufficiently informative regarding causation, because the possibility of uncontrolled or residual confounding cannot be ruled out. The fact that CVD hospitalizations in different disjoint subpopulations appear to be informative about each other suggests that these positive partial dependencies could be a result of residual confounding. 

The Bayesian network structure also indicates that PM_2.5_ is not a direct cause of CVD HAs. It shows multiple paths of association that contribute to any single measure of a C-R association (e.g., correlation or regression coefficient between PM_2.5_ and CVD hospitalizations). No single regression coefficient or other measure of association can indicate how much each path contributes to the total association. A key finding is that daily CVD HAs among older adults are dependent on variables such as county, month, year, and temperature, but conditionally independent of PM_2.5_ after conditioning on the other variables, as indicated by the fact that PM_2.5_ is not adjacent to (i.e., directly informative about) any of the CVD variables in the Bayesian network DAG models. In addition, the DAG structure indicates that the significant association observed between PM_2.5_ and CVD HAs is created by indirect paths, such as via month of the year, which is a potential confounder of PM_2.5_-CVD HA association, or via paths that involve temperature variables. The regression tree analyses also support the finding that PM_2.5_ is not a direct cause of CVD HAs.

In contrast, the potentially important distinction between statistically significant associations and evidence of causation can be lost in parametric regression models (or other association-based analyses) that do not explain the paths joining variables, but instead use a single regression coefficient (or relative risk ratio, odds ratio, attributable risk, etiologic fraction, burden-of-disease estimate, etc.) to summarize an association that may result from several distinct paths.

The findings reported here contribute primarily to the hazard identification stage of risk assessment: testing whether the datasets examined, as interpreted via nonparametric methods such as classification tree analysis and Bayesian network modeling, support the hypothesis that PM_2.5_ exerts a direct causal impact on CVD HAs that is not explained by other measured variables or by unmeasured confounders. The hypothesis of a direct causal relationship is not well supported for the data in this study; if it were, then it would be important to conduct global sensitivity and uncertainty analyses to quantify uncertainties in the causal relationship showing how CVD HA depends on PM_2.5_. The partial dependence plots in Figure 2 are a step in this direction, relying on the empirical joint distribution of the variables in the dataset (so that no parametric modeling assumptions are needed) and using a Random Forest algorithm to repeatedly sample from the set of observations and fit different classification and regression tree models to the samples—a process sometimes referred to in global sensitivity and uncertainty analysis as “bootstrapping of the modeling process” [21]. This bootstrapped nonparametric modeling gives considerable confidence that the statistical dependence of CVD HA on PM_2.5_ concentrations is at most very small, whether or not it is causal.

## 5. Conclusions

This study provides an alternative perspective for the association between air pollutants and health effects by applying several nonparametric methods to observational epidemiological data. We found that geographic location (county), time (e.g., month and year), and temperature satisfy necessary information conditions for being causes of CVD HAs among older men and women. The fact that hospitalization counts in disjoint subpopulations are strongly predictive of each other after conditioning on the aforementioned factors indicates that unobserved factors are also likely to be important causes. Taken together, our findings from nonparametric analyses do not support PM_2.5_ as a direct cause of CVD HAs among older men and women. We recommend that these and other nonparametric methods be applied to future environmental epidemiological studies so that causation can be more clearly elucidated.

## Figures and Tables

**Table 1:** Daily cardiovascular disease (CVD) hospital admissions (HAs), ambient fine particulate matter (PM_2.5_) concentrations, and meteorological factors in Dallas and Harris counties, Texas, from 2001 to 2013.

	Days with Data	Mean	SD	Minimum	10th Percentile	25th Percentile	Median	75th Percentile	90th Percentile	Maximum	Total
CVD HAs (N)											
All, 18 to 75 Years	9188	58.1	20.5	6.0	34.0	42.0	54.0	75.0	88.0	124.0	534,009
All, 75+ Years	9188	26.3	9.4	0.0	15.0	19.0	25.0	33.0	39.0	59.0	241,567
Women, 18+ Years	9188	42.3	14.7	2.0	25.0	31.0	40.0	54.0	63.0	93.0	389,072
Men, 18+ Years	9188	42.1	15.2	2.0	24.0	30.0	39.0	54.0	64.0	93.0	386,415
Women, 75+ Years	9188	16.3	6.3	0.0	9.0	12.0	16.0	20.0	25.0	43.0	149,557
Men, 75+ Years	9188	10.0	4.5	0.0	5.0	7.0	9.0	13.0	16.0	28.0	91,987
PM_2.5_ and Meteorological factors											
Daily Average PM_2.5_ Concentration (μg/m^3^)	9188	12.2	5.5	0.6	6.2	8.2	11.2	15.0	19.3	57.5	
Daily Average Temperature (°F)	9188	69.1	14.7	18.0	47.7	58.0	71.3	82.0	86.0	100.0	
Daily Minimum Temperature (°F)	9188	58.9	15.2	12.0	36.7	46.5	61.5	72.5	76.3	88.0	
Daily Maximum Temperature (°F)	9188	78.8	14.9	21.5	57.3	69.3	81.0	91.0	96.0	111.0	
Daily Dew Point Temperature (°F)	9188	55.6	15.5	5.5	32.3	44.0	60.0	68.5	72.5	77.3	

CVD = cardiovascular disease; HA = hospital admission; SD = standard deviation; PM_2.5_ = fine particulate matter; μg/m^3^ = microgram per cubic meter.

**Table 2:** Spearman correlation coefficients for PM_2.5_ and meteorological factors in Dallas and Harris counties, Texas, from 2001 to 2013.

	Daily Average PM_2.5_ Concentration	Daily Average Temperature	Daily Minimum Temperature	Daily Maximum Temperature	Daily Dew Point Temperature
Daily Average PM_2.5_ Concentration	1	0.26	0.23	0.27	0.25
		<0.0001	<0.0001	<0.0001	<0.0001
Daily Average Temperature	0.26	1	0.98	0.98	0.89
	<0.0001		<0.0001	<0.0001	<0.0001
Daily Minimum Temperature	0.23	0.98	1	0.92	0.92
	<0.0001	<0.0001		<0.0001	<0.0001
Daily Maximum Temperature	0.27	0.98	0.92	1	0.82
	<0.0001	<0.0001	<0.0001		<0.0001
Daily Dew Point Temperature	0.25	0.89	0.92	0.82	1
	<0.0001	<0.0001	<0.0001	<0.0001	

PM_2.5_ = fine particulate matter.

**Table 3:** Associations between CVD HAs and PM_2.5_ concentrations in men and women, aged 75 years and older.

Daily Counts of CVD HAs	Predictor	Linear Model ^1^		Poisson Regression Model ^1^			Quasi−Poisson Regression Model ^2^		
		Beta Coefficient ^3^	*p*-Value	Beta Coefficient ^3^	Percent Increase ^4^	*p*-Value	Beta Coefficient ^3^	Percent Increase ^4^	*p*-Value
Women, 75+	Daily PM_2.5_ Concentration	0.035 (0.017, 0.053)	0.0001	0.0021 (0.0012, 0.0031)	2.16 (1.17, 3.16)	<0.0001	0.0021 (0.00010, 0.0032)	2.16 (1.05, 3.29)	0.0001
	CVD HAs for Men (75+)	0.31 (0.29, 0.34)	<0.0001	0.018 (0.016, 0.019)		<0.0001	0.018 (0.016, 0.019)		<0.0001
Men, 75+	Daily PM_2.5_ Concentration	−0.000082 (−0.014, 0.014)	0.999	0.00015 (−0.0011, 0.0014)	0.15 (−1.1, 1.4)	0.82	0.00015 (−0.0013, 0.0015)	0.15 (−1.2, 1.56)	0.83
	CVD HAs for Women (75+)	0.19 (0.17, 0.20)	<0.0001	0.017 (0.016, 0.018)		<0.0001	0.017 (0.016, 0.019)		<0.0001

CVD = cardiovascular disease; HA = hospital admission; PM_2.5_ = fine particulate matter. ^1^ Adjusted for county, year, month, daily average temperature, minimum temperature, maximum temperature, dew point temperature, and CVD HAs for people 75 and older of the opposite sex. ^2^ Adjusted for county, year, month, daily average temperature, minimum temperature, maximum temperature, dew point temperature, CVD HAs for people 75 and older of the opposite sex, and over-dispersion. ^3^ The beta coefficients were directly estimated from each model. ^4^ The percent increases were calculated based on a 10 μg/m^3^ increment of PM_2.5_ concentration: percent increase = (exp(beta coefficient × 10) −1) × 100%.

## Supplementary Materials

The following are available online at www.mdpi.com/1660-4601/14/9/1051/s1, Figure S1: Annual average fine particulate matter (PM_2.5_) concentrations, mean temperature, dew point, and daily cardiovascular disease (CVD) hospital admissions (HAs) for adult men and women (aged 18 years and over) in Dallas County (left) and Harris County (right), Texas, 2001–2013, Figure S2: Monthly average PM_2.5_ concentrations, mean temperature, dew point, and daily CVD HAs for men and women (aged 75 years and over) in Dallas County (left) and Harris County (right), Texas, 2001–2005, Figure S3: Monthly average PM_2.5_ concentrations, mean temperature, dew point, and daily CVD HAs for adult men and women (aged 18 years and over) in Dallas County (left) and Harris County (right), Texas, 2001–2005, Figure S4: Regression tree for CVD HAs among men 75 years of age and older, Figure S5: Regression tree for CVD HAs among women 75 years of age and older, Table S1: County-specific daily CVD HAs, ambient PM_2.5_ concentrations, and meteorological factors in Dallas and Harris counties, Texas, from 2001 to 2013.
