# Supplementary material for: Applying Nonparametric Methods to Analyses of Short-Term Fine Particulate Matter Exposure and Hospital Admissions for Cardiovascular Diseases among Older Adults
Source: Int J Environ Res Public Health. 2017 Sep 12;14(9):1051. doi: 10.3390/ijerph14091051 (PMC5615588; doi:10.3390/ijerph14091051)
Supplement: Supplementary file 1 [file ijerph-14-01051-s001.pdf]

International Journal of
Environmental Research and Public Health

MDPI

Article

Applying Nonparametric Methods to Analyses of Short-term Fine Particulate Matter Exposure and Hospital Admissions for Cardiovascular Diseases among Older Adults

Louis Anthony (Tony) Cox, Jr. [1], Xiaobin Liu [2], Liuhua Shi [2], Ke Zu [2], and Julie Goodman [2,*]

[1] Cox Associates Consulting; tcoxdenver@aol.com
[2] Gradient; xliu@gradientcorp.com
Gradient; LShi@gradientcorp.com
Gradient; kzu@gradientcorp.com
Gradient; jgoodman@greadientcorp.com
* Correspondence: jgoodman@greadientcorp.com; Tel.: +1-617-395-5525

Supplementary Material

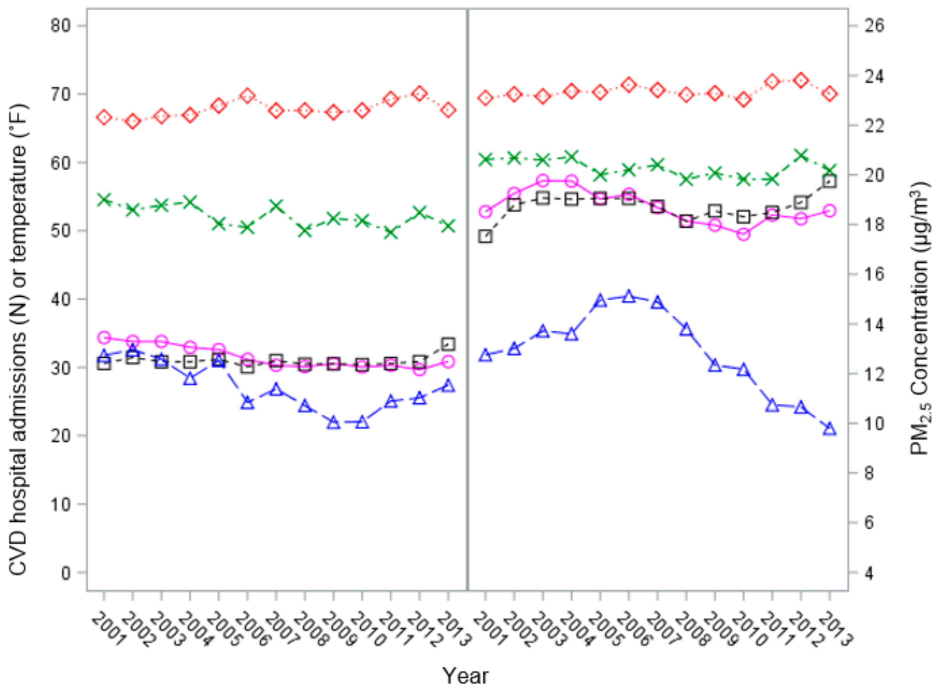

Figure S1. Annual average $PM_{2.5}$ concentrations, mean temperature, dew point, and daily CVD HAs for adult men and women (aged 18 years and over) in Dallas County (left) and Harris County (right), Texas, 2001-2013. Circles represent CVD HAs for women; squares represent CVD HAs for men; triangles represent daily $PM_{2.5}$ concentrations; diamonds represent daily average temperature; crosses represent dew point.

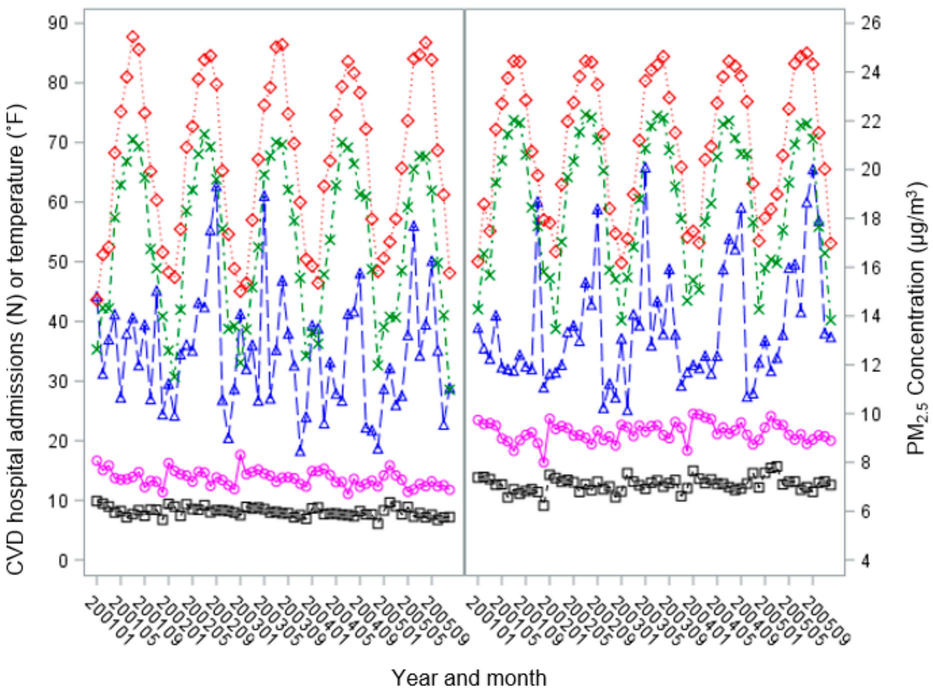

Figure S2. Monthly average $PM_{2.5}$ concentrations, mean temperature, dew point, and daily CVD HAs for men and women (aged 75 years and over) in Dallas County (left) and Harris County (right), Texas, 2001-2005. Circles represent CVD HAs for women; squares represent CVD HAs for men; triangles represent daily $PM_{2.5}$ concentrations; diamonds represent daily average temperature; crosses represent dew point.

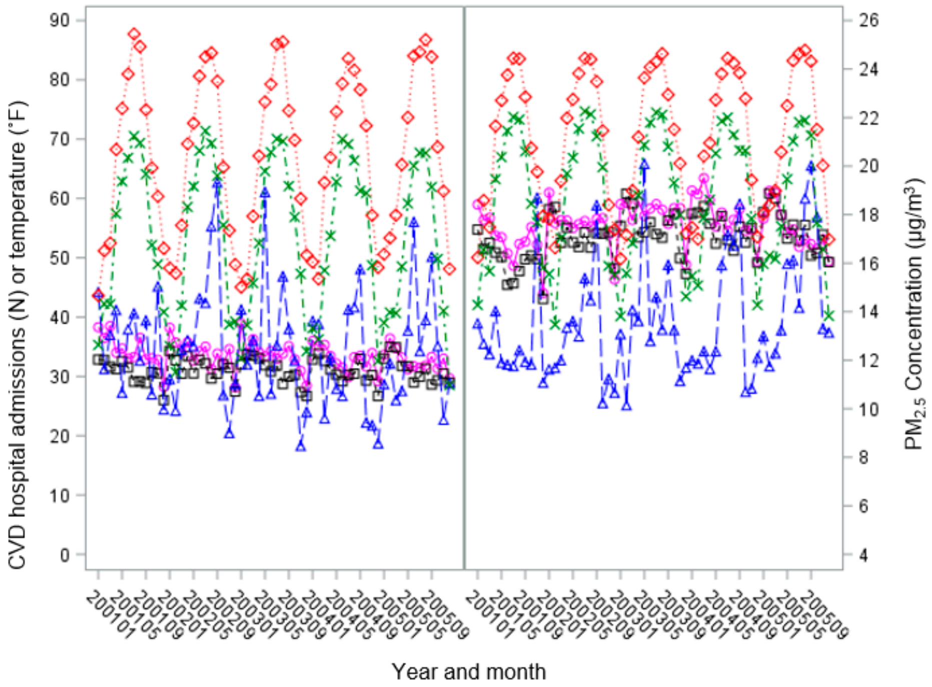

Figure S3. Monthly average $PM_{2.5}$ concentrations, mean temperature, dew point, and daily CVD HAs for adult men and women (aged 18 years and over) in Dallas County (left) and Harris County (right), Texas, 2001-2005. Circles represent CVD HAs for women; squares represent CVD HAs for men; triangles represent daily $PM_{2.5}$ concentrations; diamonds represent daily average temperature; crosses represent dew point.

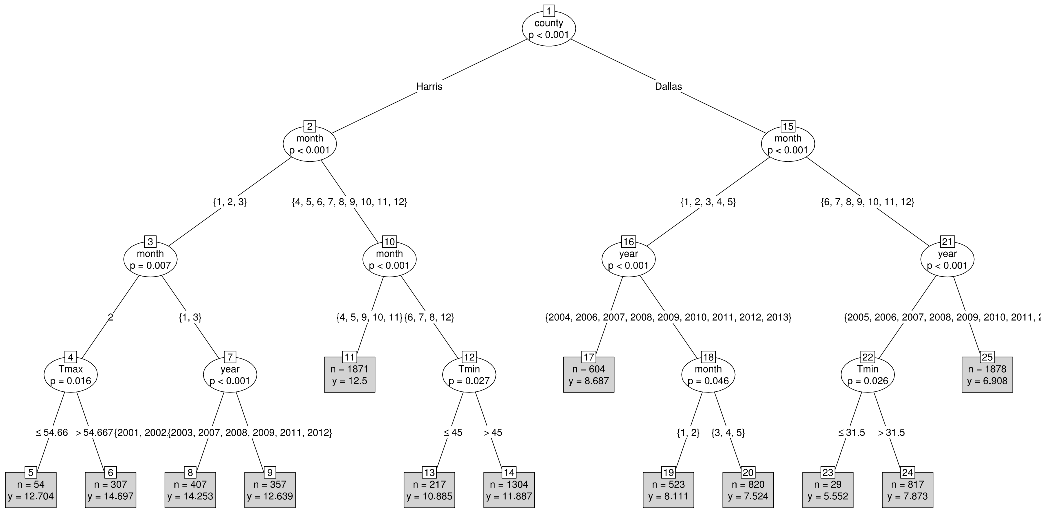

Figure S4. Regression tree for CVD HAs among men 75 years of age and older. The variables chosen by the tree-growing algorithm are identified as informative for predicting CVD HAs among older men. The intermediate nodes show the p-values from F tests for rejecting the null hypothesis that the conditional distributions are not different from each other on the left and right of each split. Each shaded "leaf" node at the bottom of the tree shows the conditional mean value of the dependent variable, given the ranges of values for the variables in the path leading to that leaf.

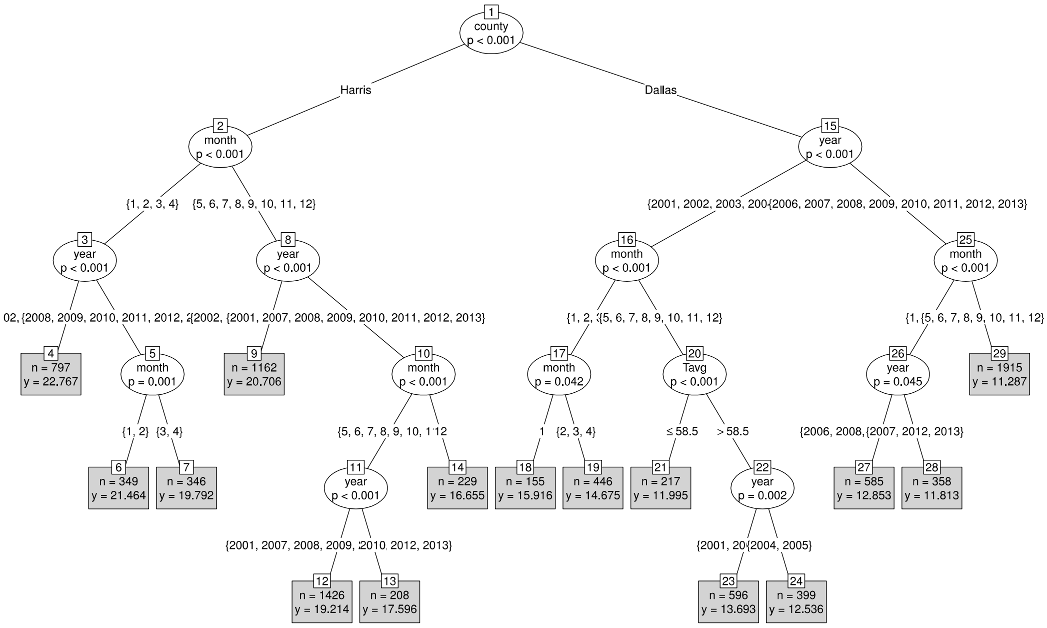

Figure S5. Regression tree for CVD HAs among women 75 years of age and older. The variables chosen by the tree-growing algorithm are identified as informative for predicting CVD HAs among older women. The intermediate nodes show the p-values from F tests for rejecting the null hypothesis that the conditional distributions are not different from each other on the left and right of each split. Each shaded "leaf" node at the bottom of the tree shows the conditional mean value of the dependent variable, given the ranges of values for the variables in the path leading to that leaf.

Table S1. County-specific daily CVD HAs, ambient $PM_{2.5}$ concentrations, and meteorological factors in Dallas and Harris counties, Texas, from 2001 to 2013

	**Days with Data**	**Mean**	**SD**	**Minimum**	**10th Percentile**	**25th Percentile**	**Median**	**75th Percentile**	**90th Percentile**	**Maximum**	**Total**
Dallas County											
CVD HAs (N)											
All, 18 to 75 years	4671	42.5	9.4	6.0	31.0	36.0	43.0	49.0	55.0	76.0	198,675
All, 75+ years	4671	20.0	5.7	0.0	13.0	16.0	20.0	24.0	27.0	42.0	93,366
Women, 18+ years	4671	31.6	7.7	2.0	22.0	26.0	31.0	37.0	41.0	63.0	147,551
Men, 18+ years	4671	30.9	7.5	2.0	21.0	26.0	31.0	36.0	41.0	58.0	144,484
Women, 75+ years	4671	12.4	4.1	0.0	7.0	10.0	12.0	15.0	18.0	31.0	58,140
Men, 75+ years	4671	7.5	3.1	0.0	4.0	5.0	7.0	10.0	12.0	22.0	35,225
$PM_{2.5}$ and Meteorological Factors											
Daily Average $PM_{2.5}$ Concentration (μg/m^3)	4671	11.5	5.4	1.6	5.8	7.7	10.5	14.2	18.4	51.2	
Daily Average Temperature (°F)	4671	67.8	16.0	18.0	45.5	55.5	69.5	81.5	87.0	100.0	
Daily Minimum Temperature (°F)	4671	57.5	16.1	12.0	34.5	44.0	59.5	72.0	77.0	88.0	
Daily Maximum Temperature (°F)	4671	77.7	16.4	21.5	54.0	66.5	79.5	91.0	97.5	111.0	
Daily Dew Point Temperature (°F)	4671	52.1	15.8	5.5	29.0	39.5	56.0	66.0	69.5	74.5	
Harris County											
CVD HAs (N)											
All, 18 to 75 years	4517	74.2	16.0	6.0	53.0	63.0	75.0	86.0	94.0	124.0	335,334
All, 75+ years	4517	32.8	8.0	1.0	23.0	27.0	33.0	38.0	43.0	59.0	148,201
Women, 18+ years	4517	53.5	11.7	4.0	39.0	45.0	54.0	62.0	68.0	93.0	241,521
Men, 18+ years	4517	53.6	12.3	4.0	38.0	45.0	54.0	62.0	69.0	93.0	241,931
Women, 75+ years	4517	20.2	5.6	1.0	13.0	16.0	20.0	24.0	28.0	43.0	91,417
Men, 75+ years	4517	12.6	4.3	0.0	7.0	9.0	12.0	15.0	18.0	28.0	56,762

	Days with Data	Mean	SD	Minimum	10th Percentile	25th Percentile	Median	75th Percentile	90th Percentile	Maximum	Total
$PM_{2.5}$ and Meteorological Factors											
Daily Average $PM_{2.5}$ Concentration (μg/m^3)	4517	12.9	5.6	0.6	6.7	8.9	11.9	15.7	20.2	57.5	
Daily Average Temperature (°F)	4517	70.4	13.2	27.7	50.7	60.7	73.0	82.0	85.3	92.3	
Daily Minimum Temperature (°F)	4517	60.4	14.0	19.0	39.3	49.0	63.7	73.0	75.7	81.3	
Daily Maximum Temperature (°F)	4517	80.0	13.1	31.7	61.0	71.7	82.0	90.7	94.7	106.0	
Daily Dew Point Temperature (°F)	4517	59.2	14.2	10.7	37.0	49.3	63.7	71.3	74.0	77.3	

CVD = cardiovascular disease; HA = hospital admission; $PM_{2.5}$ = fine particulate matter; SD = standard deviation; μg/m^3 = microgram per cubic meter.
